# Changes in pancreatic levodopa uptake in patients with obesity and new-onset type 2 diabetes: an 18F-FDOPA PET-CT study

**DOI:** 10.3389/fendo.2025.1460253

**Published:** 2025-03-03

**Authors:** Yeongkeun Kwon, Hanseok Yoon, Jane Ha, Hyeon-seong Lee, Kisoo Pahk, Hyunwoo Kwon, Sungeun Kim, Sungsoo Park

**Affiliations:** ^1^ Center for Obesity and Metabolic Diseases, Korea University Anam Hospital, Seoul, Republic of Korea; ^2^ Gut & Metabolism Laboratory, Korea University College of Medicine, Seoul, Republic of Korea; ^3^ Division of Foregut Surgery, Korea University College of Medicine, Seoul, Republic of Korea; ^4^ Division of Biotechnology, Korea University, Seoul, Republic of Korea; ^5^ Clinical and Translational Epidemiology Unit, Massachusetts General Hospital, Boston, MA, United States; ^6^ Gangneung Institute of Natural Products, Korea Institute of Science and Technology, Gangneung, Republic of Korea; ^7^ Department of Nuclear Medicine, Korea University College of Medicine, Seoul, Republic of Korea

**Keywords:** obesity, type 2 diabetes, 18 F-FDOPA PET-CT, levodopa, insulin secretion

## Abstract

**Introduction:**

Levodopa (L-3,4-dihydroxyphenylalanine)g, a dopamine precursor that circulates in the peripheral region, is involved in pancreatic glycemic control. Although previous animal studies have shown that peripheral levodopa is correlated with insulin secretion in pancreatic beta cells, the mechanism by which the pancreas uses levodopa differently in humans with obesity and type 2 diabetes remains unknown. Our study aimed to observe how the pancreas uptakes and utilizes levodopa differently under obese and diabetic conditions.

**Materials and method:**

^18^F-fluoro-L-dopa positron emission tomography-computed tomography (^18^F-FDOPA PET-CT) was used to visualize how the human body uses levodopa under obese and diabetic conditions and presented its clinical implications. 10 patients were divided into 3 groups: 1) Group A, normal weight without type 2 diabetes; 2) Group B, obese without type 2 diabetes; and 3) Group C, obese with new-onset type 2 diabetes. All patients’ lifestyle modification was conducted prior to ^18^F-FDOPA PET-CT, and plasma samples were collected to confirm changes in amino acid metabolites.

**Results:**

Pancreatic levodopa uptake increased in obese patients with insulin resistance, whereas it decreased in obese patients with new-onset type 2 diabetes [standardized uptake value (SUV) mean in participants with normal weight, 2.6 ± 0.7; SUV_mean_ in patients with obesity, 3.6 ± 0.1; SUV_mean_ in patients with obesity and new-onset type 2 diabetes, 2.6 ± 0.1, P = 0.02].

**Conclusions:**

This suggested that the alterations in the functional capacity of pancreatic beta cells to take up circulating levodopa are potentially linked to the insulin resistance and the pathogenesis of type 2 diabetes. The differences in the uptake values between the groups implied that pancreatic levodopa uptake could be an early indicator of type 2 diabetes.

## 1 Introduction

Obesity and type 2 diabetes are global health challenges intricately linked through insulin resistance and chronic low-grade inflammation. Obesity contributes to type 2 diabetes by increasing adipose tissue-derived inflammatory cytokines which impair pancreatic beta-cell function, leading to progressive glycemic dysregulation (1).

Beta cells play a central role in glucose homeostasis by secreting insulin in response to rising blood glucose, thus their function has been a therapeutic target for both obesity and type 2 diabetes (2).

Levodopa (L-3,4-dihydroxyphenylalanine) or L-DOPA circulating in the peripheral region has been revealed to influence insulin secretion and glycemic control within pancreatic beta cells (3, 4). As a precursor to dopamine, Levodopa is mostly obtained through dietary tyrosine and absorbed in the upper gastrointestinal tract (5, 6), and synthesizes dopamine (7).

The role of levodopa is widely recognized as a treatment for Parkinson’s disease due to its low molecular weight, which enables it to cross the Blood-Brain Barrier (BBB) and elevate dopamine levels in the central regions (8). In 1976, researchers discovered that long-term levodopa therapy in Parkinson’s disease patients could elevate blood glucose levels in the peripheral region (9). Combined with the hyperglycemic responses observed in rats injected with levodopa and dopamine in 1967 (10), abnormal glycemic response concerned with levodopa prompted further investigations into the relationship between the pancreatic dopaminergic pathway and glycemic control.

**Figure 1:**
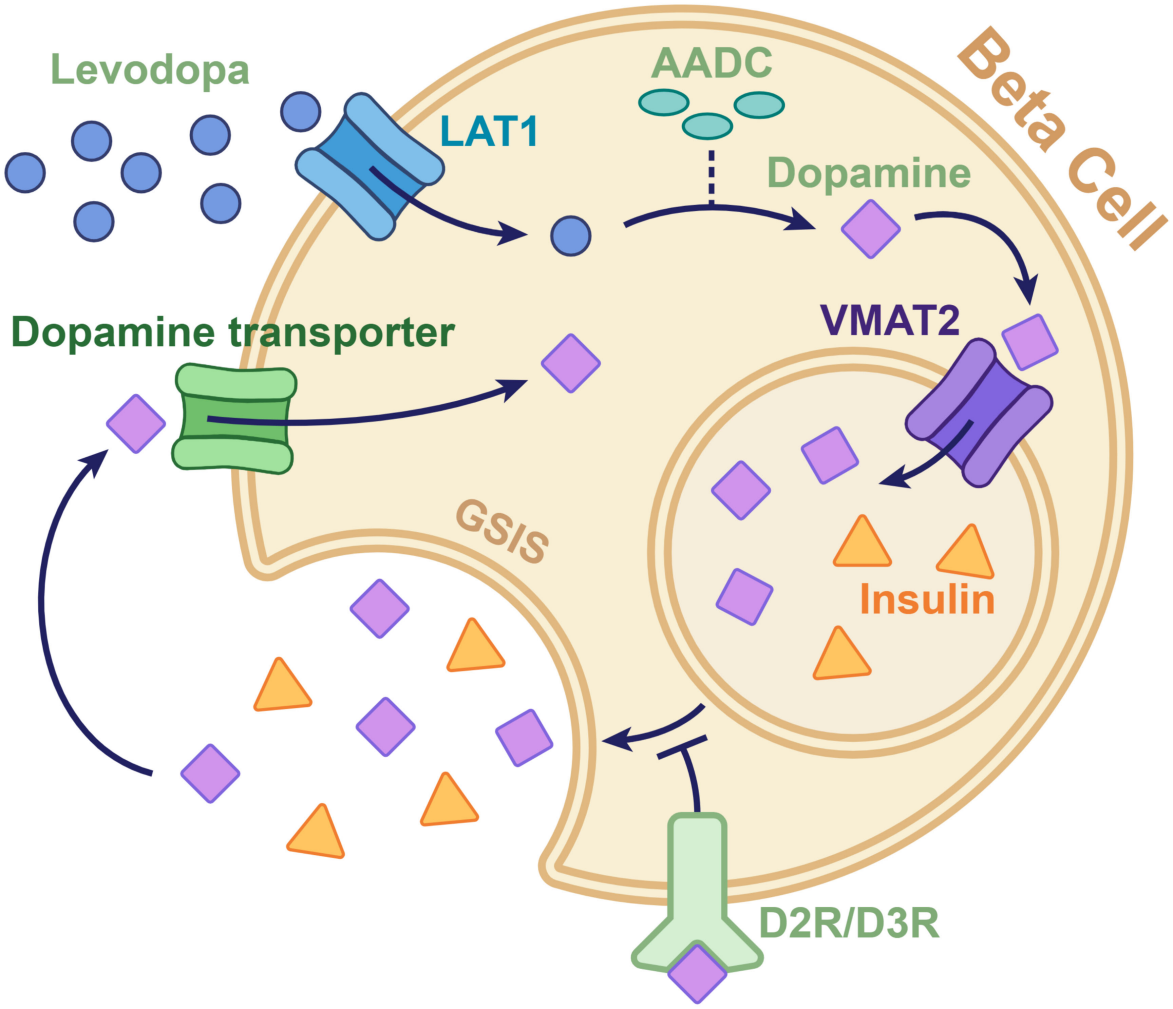
Diagram of the dopaminergic pathway involved in insulin secretion in the pancreatic beta cell. Levodopa, which is taken up by the L-type amino acid transporter 1 on the beta cell membrane, is converted to dopamine by aromatic L-amino acid decarboxylase. The converted dopamine is then transported from the cytoplasm to the intracellular vesicles by the vesicular monoamine transporter 2. The dopamine in vesicles is released through exocytosis along with insulin. The dopamine co-secreted with insulin can either be taken back into the cytoplasm by the dopamine transporters or can bind to dopamine D2 and D3 receptors to send signals that inhibit insulin secretion. AADC, aromatic l-amino acid decarboxylase; D2R/D3R, dopamine D2 and D3 receptors; GSIS, glucose-stimulated insulin secretion; LAT1, L-type amino acid transporter 1; VMAT2, vesicular monoamine transporter 2.

Compared to the levodopa-derived dopaminergic pathway in the brain, the role of peripheral levodopa in glycemic control is not well understood. After the discovery that levodopa is absorbed by beta cells in the human pancreas and synthesizes dopamine (11), numerous studies have revealed that dopamine is co-secreted with insulin and can negatively regulate glucose-stimulated insulin secretion (GSIS), proving its potential role as a therapeutic target for type 2 diabetes (12–14).

(**Figure 1**) 1). Circulating pancreatic levodopa is uptaken by the L-type amino acid transporter 1 (LAT1) on the beta cell membrane, subsequently converting it into dopamine by aromatic L-amino acid decarboxylase (AADC) (15). Intracellular dopamine is then transported into the insulin secretory vesicles by the vesicular monoamine transporter2 (VMAT2) and co-secreted with insulin (16). Co-secreted dopamine can bind dopamine receptors and inhibit GSIS by regulating Ca^2+^ oscillations or transported into beta cells by the dopamine transporter (17).

These findings show that GSIS can be further regulated by neurotransmitters such as levodopa (18, 19). Nevertheless, the precise mechanism in which levodopa uptake and dopaminergic pathways change in the human pancreas and interact with the progression of type 2 diabetes remains unclear.

Given the growing prevalence of obesity and type 2 diabetes, understanding peripheral metabolic pathways influenced by levodopa could open new perspectives for treatment. Therefore, this study aims to bridge this gap by investigating the role of levodopa in pancreatic beta-cell function across different metabolic states.

An experimental model with subjects divided into 3 groups (Group A with normal weight, Group B with obesity, and Group C with new-onset type 2 diabetes) was used to observe and visualize how levodopa uptake changes during the onset of type 2 diabetes.

This study represents the first human model investigation to compare pancreatic levodopa uptake between different metabolic states. ^18^F-fluoro-L-dopa positron emission tomography-computed tomography (^18^F-FDOPA PET-CT) was used to visualize and quantify levodopa uptake in the pancreas, and additional metabolomic analysis was conducted to quantify levodopa and related amino acid metabolites in plasma. By observing levodopa uptake and analyzing metabolomes in each group, we aim to elucidate the potential roles of levodopa in the pathogenesis of type 2 diabetes, emphasizing its significance in developing new therapeutic alternatives for type 2 diabetes.

## 2 Materials and methods

### 2.1 Study protocol

In 2020, ten patients were prospectively recruited for the study. The study population consisted of individuals aged 30–50 years, and participants were divided into three groups based on BMI and diabetes status: 1) Group A, normal weight without type 2 diabetes (n=3); 2) Group B, obese without type 2 diabetes (n=3); and 3) Group C, obese with new-onset type 2 diabetes (n=3). The male-to-female ratio of each group was 2:1 or 1:2. We enrolled an additional patient with type 1 diabetes to compare the degree of beta cell dysfunction assessed using ^18^F-FDOPA PET-CT with that of other cohorts. Obesity was defined as having a body mass index (BMI) ≥30 kg/m^2^ and normal weight as having a BMI within 18.5–25 kg/m^2^. New-onset type 2 diabetes was diagnosed in participants with a glycated hemoglobin (HbA1c) level ≥6.5%, a 2-h plasma glucose level ≥200 mg/dL in the 75-g oral glucose tolerance test, no history of type 2 diabetes or diabetes medication use, and confirmation of normoglycemia in a blood test within 2 years.

Patients were included if they had 1) BMI 18.5-25 kg/m2 and no history of type 2 diabetes (Group A); 2) BMI ≥ 30 kg/m2 and no history of type 2 diabetes (Group B); 3) BMI ≥ 30 kg/m2 and diagnosed with new-onset type 2 diabetes (Group C); 4) type 1 diabetes and beta cell dysfunction; 5) age with ≥ 30 and < 50 years old.

Patients were excluded if they had 1) type 2 diabetes who tested positive for islet cell antibodies (anti-glutamic acid decarboxylase and anti-insulin); 2) >5% change in the body weight within the last 6 months; 3) a history of medication use that could influence the results of ^18^F-FDOPA PET-CT, including carbidopa, haloperidol, monoamine oxidase inhibitors, or reserpine; 4) an estimated glomerular filtration rate <90 mL/min/1.73 m^2^; 5) previously undergone complex thoracic, abdominal, and/or pelvic surgeries; 6) chronic liver diseases; 7) gastrointestinal disorders including malabsorptive disorders or inflammatory bowel diseases; or 8) malignancy history; 9) pregnancy <. The inclusion and exclusion criteria are presented in **Supplementary Table 1**. As a pilot study, power calculation was not conducted, since no prior data was available for reference. Preliminary data, including metabolomic analyses, were internally reviewed to validate the sample’s appropriateness and ensure its relevance for exploratory analysis. Informed consent was obtained from all participants prior to the study enrollment. This study was approved by the Institutional Review Board (no. 2018AN0256).

### 2.2 Outcome measurements

The homeostatic model assessment indices were calculated using the HOMA2 calculator (20). HOMA2-S provides an estimation of insulin sensitivity, presented as percentages. The model has previously been calibrated to equate 100% with the value obtained from healthy adults. HOMA-2-IR provides an estimation of insulin resistance. Additionally, the insulinogenic index for beta cell function was computed using the formula (insulin 30 min−insulin fasting)/(glucose 30 min−glucose fasting) (21).

### 2.3 Lifestyle modification prior to ^18^F-FDOPA PET-CT

**Figure 2:**
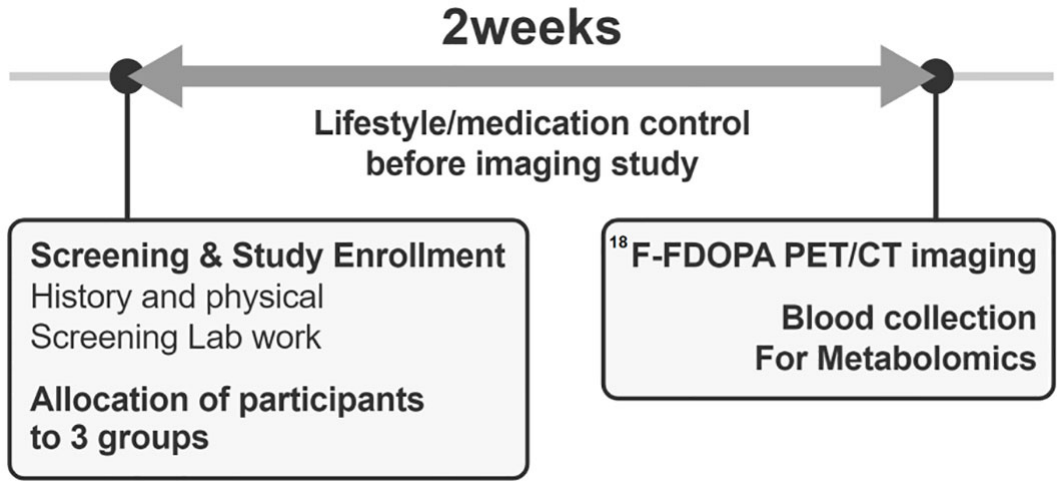
A diagram of the study progress.

After enrollment, participants underwent a 2-week pre-study optimization period to minimize the confounding effects of lifestyle or medication use (**Figure 2**). Although the dietary intake of amino acids has a minor effect on the circulating amino acid concentrations (22), to increase the reliability of the study results, participants were required to maintain protein and amino acid intake within ±20% of the recommended levels (23). They performed 150–200 min of walking exercises for 3 days per week, with a perceived exertion rate of 12–14 on the Borg scale (24). Medications influencing glucose levels or weight status were identified and discontinued before the study evaluation. Participants were monitored via phone calls and text messages to ensure adherence to the hospital-recommended lifestyle before undergoing ^18^F-FDOPA PET-CT.

### 2.4 Plasma metabolite measurements

After 2 weeks of lifestyle and medication interventions, plasma samples were collected after 8-h of overnight fasting before undergoing ^18^F-FDOPA PET-CT (**Figure 2**). Liquid chromatography-mass spectrometry was used to measure the plasma levels of the targeted amino acid metabolites (levodopa, leucine, isoleucine, phenylalanine, tryptophan, valine, and tyrosine), which are transported into the pancreatic beta cells by LAT1. The detailed protocol for measuring the plasma metabolite levels is presented in **Supplementary Table 2**.

### 2.5 ^18^F-FDOPA PET-CT imaging protocol

PET-CT was performed using a dedicated scanner (Gemini TF 16; Philips Medical Systems). All participants fasted for 8 hours before scanning, and without carbidopa premedication was administered. Patients were injected with 4 MBq/kg of ^18^F-FDOPA one hour before scanning. CT for attenuation correction was performed from the patient’s skull vertex to the upper thigh, and static PET was performed for 1 min with the patient lying supine in bed. The acquired transaxial PET/CT images were analyzed using a dedicated workstation (Extended Brilliance Workspace 4.0). The SUV was calculated using the following formula: tracer activity within the volume of interest (VOI)(MBq/mL)/injected FDOPA dose (MBq/kg body weight). The following metabolic parameters were measured: maximum and mean SUVs (SUV_max_ and SUV_mean_), dopaminergic tumor volume (DTV40), and total lesion FDOPA activity (TLDA). SUV_max_ is the value of the voxel with the highest count within a VOI. SUV_mean_ is the mean SUV value within a VOI. DTV40 was defined as a volume greater than the fixed threshold of 40% of the maximum activity within the VOI. TLDA was calculated by multiplying the SUV_mean_ with the DTV40 values.

### 2.6 Statistical analysis

In this study, the sample size was small, and the data did not meet the assumptions of normality and homoscedasticity. Therefore, non-parametric tests, including the Mann–Whitney or Kruskal–Wallis tests, were utilized to compare continuous variables between the groups. Dunn’s test was applied for *post hoc* multiple comparisons when significance was identified in the Kruskal-Wallis test. Statistical analyses were performed using the Stata 16 software (StataCorp, College Station, TX, USA). Statistical significance was defined as a two-tailed P value of <0.05.

## 3 Results

### 3.1 Characteristics of the study participants

The participants’ mean ages were 36.0 ± 5.6, 38.0 ± 8.9, and 40.7 ± 7.4 years in Groups A, B, and C, respectively (**Table 1**). The mean BMI values were 22.2 ± 1.4 kg/m^2^, 37.2 ± 2.6 kg/m^2^, and 38.5 ± 2.6 kg/m^2^ in Groups A, B, and C, respectively, demonstrating significant differences (P<0.05). The fasting plasma insulin and C-peptide levels were 7.94 ± 3.20 mIU/mL and 1.19 ± 0.33 ng/mL in Group A, 22.41 ± 1.06 mIU/mL and 3.45 ± 1.30 ng/mL in Group B, and 54.63 ± 24.07 mIU/mL and 5.89 ± 2.22 ng/mL in Group C, respectively, demonstrating significant differences (P<0.05). Group B exhibited the highest insulinogenic index (2.0 ± 0.8), which measures the level of insulin secretion in the pancreas (P<0.05). Group C exhibited the lowest HOMA2-S (16.9 ± 5.9%) and HOMA2-IR (6.4 ± 1.9) values for insulin sensitivity and resistance (P<0.05), respectively. Group C exhibited a significantly higher HbA1c level of 8.1 ± 0.9% than other groups (P<0.05). Patients with type 1 diabetes exhibited a fasting plasma C-peptide level of 0.06 ng/mL, indicating pancreatic beta cell dysfunction.

**Table 1:** Descriptive data of the study participants.

	Group A (n=3)	Group B (n=3)	Group C (n=3)	P value	Type 1 *diabetes* (n=1)
Group description	Normal weight without type 2 diabetes	Obesity without type 2 diabetes	Obesity with new-onset type 2 diabetes	NA	
Age, y	36.0 ± 5.6	38.0 ± 8.9	40.7 ± 7.4	0.022	50
Female, n	1	2	1	NA	0
Body mass index, kg/m^2^, ^*^	22.2 ± 1.4	37.2 ± 2.6	38.5 ± 2.6	0.0003	20.7
Fasting plasma glucose, mg/dL, ^*^	96.7 ± 6.5	103.0 ± 6.2	145.3 ± 15.8	0.0005	62
Fasting plasma C-peptide, ng/mL, ^*^	1.19 ± 0.33	3.45 ± 1.30	5.89 ± 2.22	0.006	0.06
Fasting plasma insulin, µIU/mL, ^*^	7.94 ± 3.20	22.41 ± 1.06	54.63 ± 24.07	0.0004	1.0
Glycated hemoglobin, %, ^*^	5.3 ± 0.3	5.6 ± 0.3	8.1 ± 0.9	0.0004	6.9
HOMA2-B, %, ^*^	82.1 ± 14.2	152.9 ± 13.2	140.5 ± 15.3	0.00003	NA
HOMA2-S, %, ^*^	109.9 ± 55.1	34.2 ± 2.1	16.9 ± 5.9	0.0003	NA
HOMA2-IR, ^*^	1.1 ± 0.4	2.9 ± 0.2	6.4 ± 1.9	0.0006	NA
Insulinogenic index, ^*^	0.7 ± 0.3	2.0 ± 0.8	0.1 ± 0.2	0.0001	NA
Disposition index, ^*^	3.0 ± 2.9	3.9 ± 2.2	0.1 ± 0.1	0.0032	NA
Serum metabolome level, µmol/L					
Levodopa	0.022 ± 0.002	0.022 ± 0.004	0.023 ± 0.012		
Leucine	115.8 ± 24.2	127.8 ± 26.9	141.5 ± 15.3		NA
Isoleucine	81.0 ± 13.9	95.7 ± 27.1	98.9 ± 2.5		NA
Phenylalanine	82.9 ± 6.2	90.9 ± 16.3	96.7 ± 17.1		NA
Tryptophan	85.6 ± 15.9	88.0 ± 13.7	92.6 ± 9.7		NA
Valine	168.8 ± 22.1	194.4 ± 48.0	204.6 ± 30.4		NA
Tyrosine	60.2 ± 2.2	77.4 ± 29.2	76.9 ± 8.4		NA

NA, not applicable.

^*^ The variables marked with an asterisk indicate significant differences between the 3 groups. (P < .05).

### 3.2 ^18^F-FDOPA PET-CT findings

**Figure 3:**
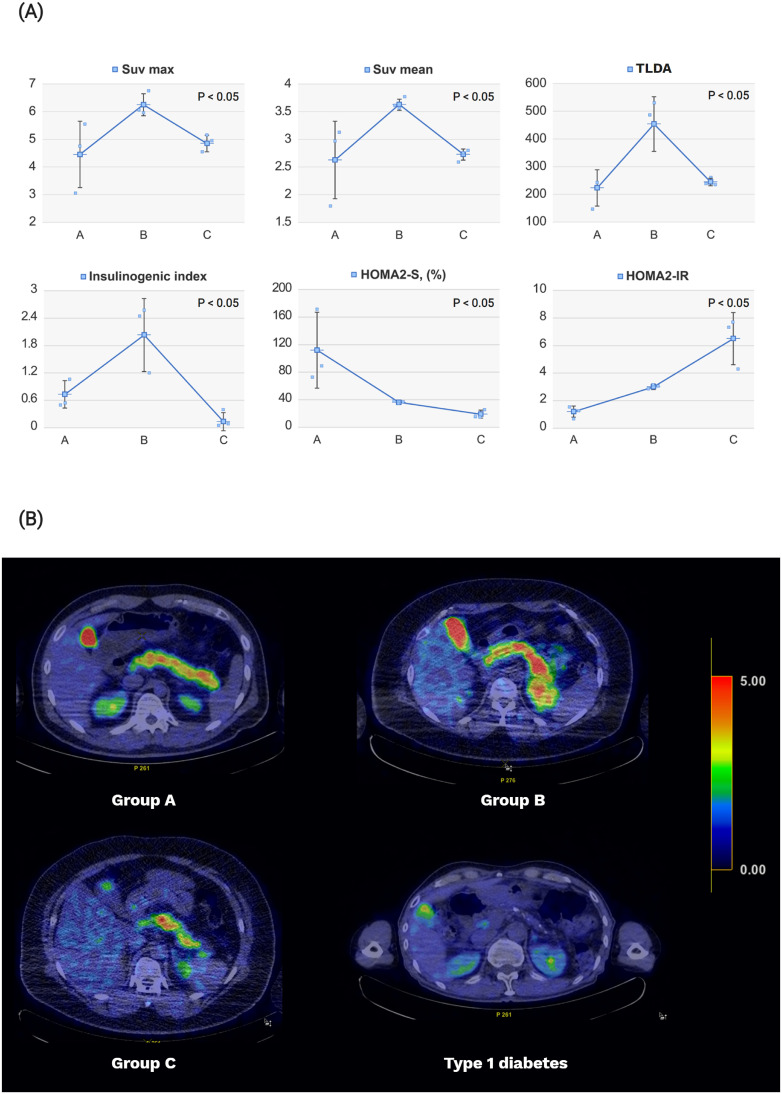
Pancreatic levodopa uptake on ^18^F-FDOPA PET-CT imaging and diabetes-related indices according to the study groups. **(A)** Obese patients (Group B) showed increases in pancreatic beta cell function represented by the insulinogenic index and pancreatic levodopa uptake compared with that of normal controls (Group A). Patients with new-onset type 2 diabetes (Group C) showed decreases in beta cell function and pancreatic levodopa uptake compared with those in Group **(B)** The degree of insulin sensitivity and insulin resistance gradually worsened in Groups A–C. The pancreatic beta cell function was expressed using the insulinogenic index, while the pancreatic levodopa uptake was expressed using the SUV_mean_, SUV_max_, and TLDA. The insulin sensitivity and insulin resistance were expressed using the HOMA2-S and HOMA2-IR values, respectively. **(B)** The ^18^F-FDOPA PET/CT image was reconstructed by changing the color according to the values of SUV_mean_. The higher the SUV_mean_ value, the closer the color to red; the lower the SUV_mean_ value, the closer the color to blue. The averages of SUV_mean_ for each group are as follows: [mean (standard deviation): Group A, 2.6 (0.7); Group B, 3.6 (0.1); and Group C, 2.6 (0.1); type 1 diabetes, 0.8]. SUV, standardized uptake value; TLDA, total lesion FDOPA activity; HOMA, homeostatic model assessment.

Group B exhibited the highest SUV_max_ and SUV_mean_ assessed from the ^18^F-FDOPA PET-CT images (SUV_max_=6.2 ± 0.4, P=0.02 and SUV_mean_=3.6 ± 0.1, P=0.02) (**Figure 3**). Group B exhibited significantly higher TLDA than Groups A or C (Group B= 448.4 ± 98.8, P=0.02) each. Patients with type 1 diabetes exhibited SUV_max_, SUV_mean_, and TLDA values of 1.0, 0.8, and 3.2, respectively.

### 3.3 Targeted metabolome data

No significant differences were observed in the plasma concentrations of the seven metabolites (levodopa, leucine, isoleucine, phenylalanine, tryptophan, valine, and tyrosine) transported by the LAT1 among the three groups (**Table 1**).

## 4 Discussion

### 4.1 Summary

We aimed to observe how levodopa uptake changes between subjects with normal weight, patients with obesity, and patients with new-onset type 2 diabetes. The ^18^F-FDOPA PET-CT and metabolomics data revealed that Group B exhibited increased pancreatic beta cell function and levodopa uptake compared to Group A. Conversely, Group C exhibited decreased pancreatic beta cell function and levodopa uptake compared to Group B. To exclude other metabolites’ involvement in insulin secretion, additional metabolite analysis was conducted and showed that changes in other metabolite uptake were insignificant among the groups. Therefore, it is less likely that changes in the uptake of other metabolites affect the function of beta cells.

The results of the ^18^F-FDOPA PET-CT and targeted metabolome data showed that levodopa uptake and beta cell function exhibited a positive correlation with varying degrees in each group. Thus, our finding calls into question the previous consensus that pancreatic dopamine inhibits glucose-stimulated insulin secretion (GSIS) through autocrine and paracrine mechanisms, suggesting that the dopaminergic pathway in pancreatic beta cells not only inhibits GSIS but also may play an additional role in modifying beta cell function. We hypothesize that changes in levodopa uptake accompany modifications in the dopaminergic pathway, contributing to changes in beta cell function. A systematic review of previous research on dopaminergic pathways in obesity and type 2 diabetes was conducted to examine modifications in pancreatic dopaminergic pathways and their implications for the development of type 2 diabetes.

### 4.2 Dopaminergic pathway modification in patients with obesity

Patients with obesity are generally known to exhibit low-grade inflammation and hyperinsulinemia (25), and our study similarly observed increased insulin secretion and higher levodopa uptake in Group B compared to Group A. The elevated levodopa uptake can be explained by enhanced activity of LAT1 in patients with obesity. Patients with obesity exhibit a higher amount of adipose tissue compared to normal-weight subjects and have beta cells in which LAT1 activity is elevated (26). This elevation is due to secretomes secreted by the adipose tissue, which cause inflammation and increase the uptake of circulating levodopa into pancreatic beta cells. Increased levodopa uptake promotes dopamine synthesis, raises intracellular dopamine levels in beta cells, and enhances insulin secretion (27). The increase in insulin secretion can be explained by an overexpression of VMAT2 (28). Overexpression of VMAT2 sequesters the elevated cytoplasmic dopamine into insulin-containing vesicles more frequently (29). Consequently, it facilitates the co-secretion of dopamine and insulin in beta cells. In summary, higher activity of LAT1 and overexpression of VMAT2 in patients with obesity can temporarily enhance insulin secretion. Hyperinsulinemia allows patients with obesity to maintain normal glycemia, which can lower blood glucose levels and prevent vascular damage.

### 4.3 Dopaminergic pathway modification in patients with type 2 diabetes

In contrast, patients in Group C demonstrated reduced insulin secretion and pancreatic levodopa uptake compared to Group B. This observation is consistent with the pathophysiology of type 2 diabetes, which is characterized by insulin resistance and beta cell dysfunction, primarily characterized by insulin underproduction (30, 31). Patients with type 2 diabetes exhibit opposite changes in dopaminergic pathways compared to Group B. In contrast to Group B, the patient model with type 2 diabetes was found to have underexpression of LAT1 in the hyperglycemic condition (32). Underexpression of LAT1 leads to a lower amount of levodopa uptake into the pancreatic dopaminergic pathway and is accompanied by the downregulation of VMAT2 (33). As VMAT2 transports dopamine to the secretory vesicles for insulin secretion, downregulation of VMAT2 activity results in decreased insulin secretion with impaired GSIS. Studies on the differential activity of the two transporters in the dopaminergic pathways under obese and type 2 diabetic conditions support our findings of different levodopa uptake among the three groups.

### 4.4 Role of dopaminergic pathway modification

As type 2 diabetes progresses and beta cells lose their function, changes in the dopaminergic pathway may serve a different role than increasing insulin secretion. Some studies have addressed the beneficial role of dopamine accumulation and increased expression of VMAT2 in obesity. Modifying the dopaminergic pathway in obese conditions can be a self-defense mechanism of the pancreas and beta cells from inflammation and oxidative stress (29). Compared to normal-weight subjects, M1-type adipose tissue macrophages are specifically present in patients with obesity and induce an inflammatory response by secreting pro-inflammatory adipokines such as interleukin- 6, TNF-α, and nitric oxide (34, 35). Accumulation of pancreatic dopamine can protect the pancreas against inflammation by reducing pro-inflammatory cytokines and microvascular permeability (36, 37). In this case, the hyperactivity of LAT1 enhances the levodopa uptake by beta cells, facilitating dopamine accumulation. Simultaneously the overexpression of VMAT2 protects pancreatic beta cells from oxidative stress caused by dopamine accumulation in the cytoplasm (38). However, little research has been conducted to show the clear implication of changes in the dopaminergic pathways in obese and type 2 diabetic patients.

### 4.5 Limitations

The limitations of this paper include a small study sample, cross-sectional design, presence of artefacts and species specificity.

Due to the exploratory nature of this pilot study, a small sample size of n=3 per group was employed to collect preliminary data and generate hypotheses for further research. While we acknowledge that the small sample size may limit the generalizability of our findings, we adopted several measures to enhance the reliability of the results, including a lifestyle/medication control period prior to ^18^F-FDOPA PET/CT, strict patient enrollment criteria, and confirmation of preliminary data, including the metabolome of participants. Despite these efforts, we fully recognize that sample size is small for human studies, particularly in the context of investigating complex metabolic pathways such as the pancreatic dopaminergic pathway. This limitation arises primarily from the resource-intensive nature of ^18^F-FDOPA PET-CT imaging and the challenges of patient recruitment under strict inclusion/exclusion criteria. However, the findings from this study serve as proof of concept for the feasibility of using ^18^F-FDOPA PET-CT in visualizing pancreatic levodopa uptake and its correlation with beta-cell function. These results can guide the design of future large-scale cohort studies, which are currently underway in our lab to confirm and expand upon the current findings.

We also acknowledge that the cross-sectional design may limit the ability to infer causality. Therefore, we propose a future longitudinal study to track pancreatic levodopa uptake over time, particularly during the transition from obesity to type 2 diabetes.

Another limitation of this study is the potential presence of artefacts in ^18^F-FDOPA PET-CT imaging. Artefacts, such as partial volume effects and motion-related distortion, can occur in PET-CT scans, particularly when imaging small organs like the pancreas. These artefacts may lead to inaccurate quantification of levodopa uptake. To minimize this risk, we applied strict imaging protocols and validated our findings through additional metabolomic analysis, which showed consistent results across groups. Despite these efforts, the possibility of artefacts cannot be entirely excluded. Therefore, future studies employing higher-resolution imaging techniques and larger cohorts are warranted to further confirm our results and rule out potential artefactual influences.

The study of pancreatic dopaminergic pathways has a crucial limitation of species specificity (39, 40), which makes generalization difficult. As prior studies were mostly conducted with different animal models, the results are difficult to generalize and may not be directly applicable to humans. Therefore, novel radiologic markers or stem cell-derived organoids can be excellent alternatives, as active research in human models is highly needed to shed light on the role of pancreatic dopamine. To overcome the problem of species specificity, our study used ^18^F-FDOPA PET-CT to investigate the pancreatic dopaminergic pathway in the human pancreas.

## 5 Conclusion

This study provides exploratory insights into the role of pancreatic levodopa uptake in obesity and type 2 diabetes. Patients with obesity exhibited increased pancreatic levodopa uptake and beta-cell function compared to normal controls, while those with obesity and new-onset type 2 diabetes showed reduced uptake and impaired beta-cell function.

Using ^18^F-FDOPA PET/CT, this is the first study to visualize changes in pancreatic levodopa uptake in human models, highlighting its decline with the onset of type 2 diabetes. These findings suggest that pancreatic levodopa uptake may serve as an early marker for type 2 diabetes and a potential therapeutic target for preserving beta-cell function.

As there are no reliable methods to measure human pancreatic beta cell mass (BCM) *in vivo* (41), ^18^F-FDOPA PET/CT can allow early detection and prevention of type 2 diabetes in the labile group by tracking levodopa absorption and visualizing the overall function of beta cells in a minimally invasive manner (42).

Currently, there are many unknowns due to the lack of research on the dopaminergic pathway in the peripheral region. Previous studies have reported that levodopa uptake reduces glucose-stimulated insulin secretion (GSIS) through autocrine or paracrine mechanisms in animal models. However, our findings suggest that increased levodopa uptake may transiently stimulate insulin secretion, co-released with dopamine, particularly in individuals with obesity but without diabetes. This apparent discrepancy is less likely due to species-specific differences between animal models and humans, and instead may reflect short-term versus long-term effects of levodopa uptake on beta-cell function. Specifically, levodopa uptake may temporarily promote dopamine synthesis and insulin secretion (43), while chronic exposure or overexpression of dopaminergic signaling may inhibit GSIS, as observed in previous studies.

Additionally, studies on how dopamine affects pancreatic glucagon secretion and stimulates insulin secretion in a concentration-dependent manner suggest that the dopaminergic pathway is complex research target than previously thought (44). Therefore, further investigations are required to estimate the precise mechanism of the dopaminergic pathway in the peripheral region. Our study is meaningful for observing changes in levodopa uptake in the human pancreas during the onset of type 2 diabetes and for highlighting the potential processes and roles of modification in the pancreatic dopaminergic pathway based on previous research.

Given the pilot study’s small sample size, further research with larger cohorts is necessary to validate these findings and establish the clinical relevance of pancreatic dopaminergic pathways. Future studies should also explore the dual role of dopamine in regulating insulin and glucagon secretion, as well as its broader implications for metabolic health. Despite its limitations, this study provides a foundation for understanding the dynamic role of levodopa in the pancreas and its potential impact on the prevention and management of type 2 diabetes.

## Data Availability

The original contributions presented in the study are included in the article/**Supplementary Material**. Further inquiries can be directed to the corresponding authors.
