# Supplementary material for: Changes in pancreatic levodopa uptake in patients with obesity and new-onset type 2 diabetes: an 18F-FDOPA PET-CT study
Source: Front Endocrinol (Lausanne). 2025 Mar 3;16:1460253. doi: 10.3389/fendo.2025.1460253 (PMC11911206; doi:10.3389/fendo.2025.1460253)
Supplement: Supplementary file 1 [file DataSheet1.docx]

**Supplemental Table 1.** Inclusion and exclusion criteria

| Inclusion criteria | 1. BMI 18.5-25 kg/m2 and no history of type 2 diabetes (Group A), BMI ≥ 30 kg/m2 and no history of type 2 diabetes (Group B), BMI ≥ 30 kg/m2 and diagnosed with new-onset type 2 diabetes (Group C), and additional one patient with type 1 diabetes for the comparison of beta cell dysfunction  2. ≥ 30 and < 50 years old |
| --- | --- |
| Exclusion criteria | 1. Positive for islet cell antibody (e.g. anti-GAD, anti-insulin) in patients with type 2 diabetes  2. Change more than 5 % of the total body weight in the last 6 months  3. Any medication history such as carbidopa, haloperidol, monoamine oxidase inhibitors, or reserpine that could influence 18F-FDOPA PET-CT imaging  4. Estimated glomerular filtration rate (eGFR) < 90 ml/min/1.73m2  5. Prior complex thoracic, abdominal, and/or pelvic surgery includging stomach, small intestine, large intestine, bile duct, pancreas, spleen  6. Chronic liver diseases including liver cirrhosis and chronic hepatitis  7. Gastrointestinal disorders including malabsorptive disorders or inflammatory bowel diseases  8. Malignancy history  9. Pregnancy  10. Any condition or major illness that in the investigator’s judgment places the subject at undue risk by participating in the study |
| Abbreviations: BMI, body mass index  New-onset type 2 diabetes was diagnosed if the glycated hemoglobin (HbA1c) ≥ 6.5 % and the two-hour serum glucose ≥ 200 mg/dL in 75g oral glucose tolerance test, and participants had no history of type 2 diabetes or taking diabetes medications, and normoglycemia was confirmed in blood test within 2 years. | |

**Supplemental Table 2.** UPLC-Q Exactive Orbitrap MS analysis, and data processing for quantification

| 1. A 5 μL of internal standards (IS) mixtures spiked to 200 μL of serum samples, respectively. Subsequently, a 700 μL of cold methanol (MeOH) was added for protein precipitation, and centrifuged at 12,300 g for 10 min at 4℃ after vortex-mix for 30 sec. Next, 800 μL of the supernatant was dried using a SpeedVac vacuum Concentrator (Thermo Fisher Scientific, Waltham, MA, USA), respectively, and re-dissolved in 100 μL of 0.1% formic acid in water. Then, 10 μL of the samples were injected into an UPLC-Q Exactive Orbitrap MS system (Thermo Fisher Scientific, Waltham, MA, USA). 2. Chromatographic separation was achieved using UPLC system (Vanquish UHPLC, Thermo Fisher Scientific, Waltham, MA, USA) with Waters ACQUITY UPLC® HSS T3 (50 x 2.1 mm, 1.8 μm, 100 Å) with ACQUITY UPLC® HSS T3™ VanGuard Pre-Column (2.1 x 5 mm, 1.8 μm). The mobile phase was composed of solvent A (0.1% formic acid in LC-MS grade MeOH with 5% LC-MS grade Water (*v/v*)) and solvent B (0.1% formic acid in LC-MS grade Water with 5% LC-MS grade MeOH (*v/v*)). The total flow rate and column temperature were set a 0.4 mL/min and 35℃, respectively. The gradient program of the mobile phase was set to initially 0% B (0 min), increased to 20% B (0-2 min), increased to 100% B (2-5 min) and then held at 100% B (5-10 min), and decreased to the initial 0% B, followed by a 5 min re-equilibration period. 3. Q Exactive Orbitrap MS was operated with equipped heated electrospray ionization (H-ESI II) probe in the parallel reaction monitoring (PRM) mode. Resolution was set at 35,000. Automatic gain control, maximum injection time were set at 3e6 and 100 ms, respectively. Normalized collision energy was set to 50%, isolation window was set at 1.4 m/z. In the H-ESI II probe setting with positive ion mode, the spray voltage was set at 3.5 kV, the capillary temperature and aux gas heater temperature were set at 300 and 400℃, respectively, sheath gas and aux gas flow rate were set at 40 and 10, respectively, and the S-lens rf level was 55. 4. The MS data was processed to quantification using Skyline. In transition settings, mass tolerance set at 0.01 m/z. |
| --- |
